# *Beauveria bassiana* Water Extracts’ Effect on the Growth of Wheat

**DOI:** 10.3390/plants12020326

**Published:** 2023-01-10

**Authors:** Dawid J. Kramski, Daria Nowinski, Kaja Kowalczuk, Piotr Kruszyński, Jagoda Radzimska, Beata Greb-Markiewicz

**Affiliations:** 1 Department of Advanced Material Technology, Faculty of Chemistry, Wroclaw University of Science and Technology, Wybrzeze Wyspianskiego 27, 50-370 Wroclaw, Poland; 2 Department of Analytical Chemistry and Chemical Metallurgy, Faculty of Chemistry, Wroclaw University of Science and Technology, Wybrzeze Wyspianskiego 27, 50-370 Wroclaw, Poland; 3 Students Science Association Bio-Top, Faculty of Chemistry, Wroclaw University of Science and Technology, Wybrzeze Wyspianskiego 27, 50-370 Wroclaw, Poland; 4 Department of Organic and Organic and Medicinal Chemistry, Faculty of Chemistry, Wroclaw University of Science and Technology, Wybrzeze Wyspianskiego 27, 50-370 Wroclaw, Poland; 5 Department of Biochemistry, Molecular Biology and Biotechnology, Faculty of Chemistry, Wroclaw University of Science and Technology, Wybrzeze Wyspianskiego 27, 50-370 Wroclaw, Poland

**Keywords:** entomopathogenic fungi, food security, fertilizer, micronutrients, organic matter, plant growth biostimulants, sustainability

## Abstract

For a long time, entomopathogenic fungi were considered alternative biological control factors. Recently, these organisms were shown to fulfill additional roles supporting plants’ development, improving their resistance to disease and survival under stress conditions. Considering the documented interactions of *B. bassiana* with a wide range of plants, we aimed to evaluate the impact of aqueous extracts of the fungus on the growth of an agriculturally significant plant—wheat. The usage of fungal extracts instead of fungi could be beneficial especially in unfavorable, environmentally speaking, regions. Selected dilutions of the crude extract obtained under different pH and temperature conditions were used to establish the optimal method of extraction. Plant growth parameters such as length, total fresh weight, and chlorophyll composition were evaluated. Additionally, the antibacterial activity of extracts was tested to exclude negative impacts on the beneficial soil microorganisms. The best results were obtained after applying extracts prepared at 25 °C and used at 10% concentration. Enhancement of the tested wheat’s growth seems to be related to the composition of the extracts, which we documented as a rich source of macro- and microelements. Our preliminary results are the first confirming the potential of fungal water extracts as factors promoting plant growth. Further detailed investigation needs to be carried out to confirm the effects in real environment conditions. Additionally, the consistency of the plant growth stimulation across different entomopathogenic fungi and agriculturally used plant species should be tested.

## 1. Introduction

The population of the world is predicted to increase to approximately 9.1 billion by 2050 [1]. Considering this prediction, the Food and Agriculture Organization (FAO) of the United Nations published a report discussing the future of food and agriculture. In the report, the need for further development of alternative, efficient, and sustainable methods to produce food and other non-food agricultural goods was presented [2]. The alternative method of agricultural production, limiting the use of synthetic pesticides harmful to natural resources, depends on the use of biological factors including entomopathogenic fungi [3]. The first study on *Beauveria bassiana* infecting silkworm culture was performed in the early 1800s. To date, more than 700 species from approximately 90 different genera have been shown to be insect-pathogenic fungi. However, the most frequently studied and currently commercially used are strains belonging to the genera *Beauveria, Metarhizium, Isaria, Hirsutella, and Lecanicillium* [4].

### 1.1. Entomopathogenic Fungi as Plant-Growth-Improving Agents

The application of inorganic fertilizers in the last century has increased agricultural production by about 50%; however, it has also led to deterioration of the environment. For this reason, development of new alternatives in the form of naturally obtained, environmentally friendly and cost-effective biofertilizers is advantageous. Biofertilizers consist of microorganisms such as bacteria or fungi with the capacity to add nutrients to the soil, fixing nitrogen and ensuring solubilization of minerals to be used by the plant [5,6]. Recently, the ability of entomopathogenic fungi to act as endophytes and to improve nutrient uptake and plant growth was presented [7,8]. This discovery showed additional benefits associated with the use of these organisms. The term endophyte was used for the first time by De Bary (1866) to define microorganisms that occur within the plant tissues without causing any harm to the plants [9]. In addition to endophytic interactions, entomopathogenic fungi were shown to form mycorrhiza-like interactions and take part in the response to stress (both biotic and abiotic) and absorption of water and nutrients [8]. Both endophytic and mycorrhiza-like interactions usually are beneficial for plants [10]. It was shown that maize seeds treated with *B. bassiana* grew as higher plants with longer roots presenting higher biomass [11]. Additionally, inoculation of bean seeds in conidial suspensions of *B. bassiana* and *M. robertsii* caused improvement in plants’ growth in comparison to the control. Treatment improved not only plants’ length, but also the number of leaves and the fresh and dry weight of roots [12]. *B. Bassiana* was also shown to influence plant metabolism by increasing the level of total alkaloids in tested chive [13].

During plant growth, the availability of elementary nutrients is crucial. Nitrogen constitutes 78% of the atmosphere in a form that it is unavailable to plants unless it is fixed by microbial symbionts or free-living bacteria. For this reason, nitrogen is a limiting nutrient in many natural and agricultural settings. Entomopathogenic fungi were shown to translocate to plant nitrogen acquired from insect material [14]. Another essential element is iron, being a component of many enzymatic systems and taking part in major processes, such as photosynthesis. Studies documented improvement in iron availability in the presence of endophytic *B. bassiana* and *M. brunneum* leading to an increase in leaf chlorophyll content and the length of roots [15]. A tissue analysis of grapevine infected by *B. bassiana* revealed significantly increased contents of calcium and magnesium [16]. *M. brunneum* was shown to increase the uptake of manganese and zinc. Though the obtained results were dependent on the crop species, type of soil, time, and method of fungus application, the research confirmed the need and the advisability of studying the use of entomopathogenic fungi as new promising agents for crop production [17].

### 1.2. Entomopathogenic Fungi as Plant Disease and Stress Control Agents

Entomopathogenic fungi supporting plants’ development were shown to improve their resistance to disease and survival under stress conditions [18]. For example, a cauliflower plant treated with *B. bassiana* spores presented a different profile of secondary metabolites and increased resistance to *Plutella xylostella* in comparison to untreated cauliflower. It was shown that *P. xylostella* larvae were not able to develop on the plant treated with *B. bassiana* leaves. [19]. Hwi-Geon Yun et. al. revealed that isolates from *B. bassiana* and *M. anisopliae* display activity against *Myzus persicae* and *Botrytis cinerea* [20]. *B. bassiana* produces a wide range of secondary metabolites, including beauvericin, bassianolides, oosporein, cyclosporin A, and oxalic acid, presenting cytotoxic, antibacterial, and antifungal activities [21]. The presence of *B. bassiana* induced the production of the following volatile compound by melon and cotton: benzaldehyde, (*2Z,13E*)-octadeca-2,13-dien-1-ol, and hexadecan-4-yl 2,2,2-trifluoroacetate [22].

Plants, as living organisms, exhibit various mechanisms of defense against parasites and pathogens, which can be described as age-related resistance, organ-specific resistance, and induced resistance (IR), which is a resistance triggered by activation of genetically programmed pathways inside plants to diminish the effect of consecutive pathogen attacks [23]. Plants exhibit two forms of induced resistance: systemic acquired resistance (SAR) and induced systemic resistance (ISR). In comparison to SAR acting by protein accumulation and the salicylic acid pathway, ISR relies on pathways regulated by jasmonate and ethylene [24]. Interestingly, genome-wide expression analysis of *A. thaliana*, whose roots were dipped in *B. bassiana* conidial suspension, provided evidence for transcriptional reprogramming, also explaining the observed resistance against phytopathogen *Sclerotinia sclerotiorum*. Root colonization caused by *B. bassiana* strains caused strain-specific changes in the expression of genes related to pathogenesis, phytoalexins, jasmonate, and salicylic acid pathways [25]. Additionally, inoculation of cabbage with *M. brunneum* resulted in an increase in jasmonate and salicylic acid production in parts of the plant that were colonized by the fungus [26].

Considering the antagonistic activity of entomopathogenic fungi against insects and plant diseases in addition to their beneficial effect on nutrient uptake, it would be highly beneficial to develop a method for their use in sustainable agricultural production.

Our study aimed to test the effect of *B. bassiana* water extracts on the growth of wheat seedlings. It is important to mention that present study is the first trial to evaluate the suitability of entomopathogenic fungi extracts as factors improving the growth of agriculturally important plants. Selected dilutions of the crude extract obtained under different pH and temperature conditions were used to establish the optimal method of extraction. Plant length, total fresh weight, and chlorophyll composition, in addition to micro- and macroelements and toxic metals, were evaluated. Additionally, the antibacterial activity of extracts was tested to exclude their negative impact on the beneficial soil microorganisms.

## 2. Results

### 2.1. Effect of B. bassiana Water Extracts on the Growth of Wheat Seedlings

**Figure 1:**
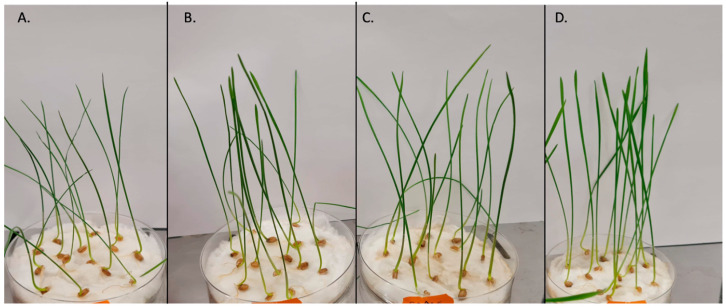
Wheat seedlings after 14 days of incubation. The control group (**A**) was watered only with water, while the other groups were treated with water fungal extracts obtained at 75°C: (**B**) pH = 7, concentration 0.5%; (**C**) pH = 3, concentration 2.5%; (**D**) pH = 10, concentration 0.5%.

The effect of the fungal extracts on the growth of the tested wheat was evaluated via the germination test (Figure 1). After 14 days, the plants from each group were harvested. Then, their total length and fresh weight were determined.

#### 2.1.1. Length of Cultivated Wheat

**Figure 2:**
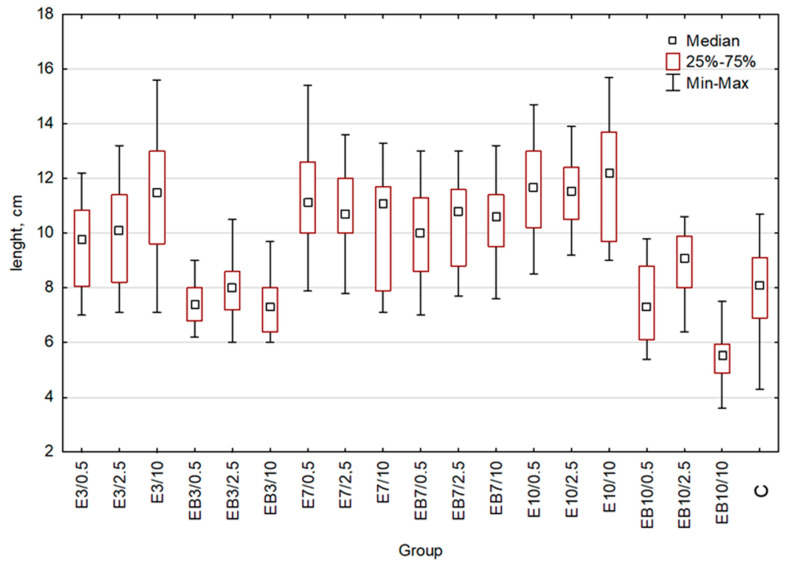
Wheat length comparison: whiskers represent the maximum and minimum, the box represents interquartile range (25–75%), square denotes the median for the entire sample. C-control, E3-group treated with the aqueous extract obtained at 25 °C pH = 3, E7-group treated with the aqueous extract obtained at 25 °C pH = 7, E10-group treated with the aqueous extract obtained at 25 °C pH = 10, EB3-group treated with the aqueous extract obtained at 75 °C pH = 3, EB7-group treated with the aqueous extract obtained at 75 °C pH = 7, EB10-group treated with the aqueous extract obtained at 75 °C pH = 10. Concentrations used: 0.5-concentration 0.5%, 2.5-concentration 2.5%, 10-concentration 10%.

The length of the plant was one of the growth parameters. To test and compare the efficiency of obtained extracts as stimulants of wheat growth, we treated each group of wheat seedlings (20 plants for each group) with extract obtained at 25 or 75 °C and pH 3, 7, or 10, in 3 dilutions of extracts (0.5%, 2.5%, and 10%). As the control, we used the group of plants treated with water. After harvesting, plant length was measured (Figure 2 and Table 1). An indication of statistically significant differences between the studied groups is shown in Table 1. Additional data important for statistical analysis are presented in the Supplementary Materials (Tables S1–S3). For aqueous extracts obtained at 25 °C and pH = 3 or pH = 10 (E3 and E10), we observed the increasing of plant length proportionally to the extract concentration, similarly to EB7 extracts (aqueous extract obtained at 75 °C pH = 7). Interestingly, in the case of E7 extracts (25 °C, pH = 7), the increase in concentration resulted in a diminished length of plants. Treatment of plants with EB3 and EB10 extracts (EB3—aqueous extract obtained at 75 °C pH = 3, EB10—aqueous extract obtained at 75 °C pH = 10) resulted in a diminished length of wheat seedlings for all used concentrations. The highest reduction was observed for the highest concentration (10%) of extracts, especially the use of the EB10 extract, which presented a negative effect on plant growth (plants’ length decreased by 32% in comparison to the control group).

#### 2.1.2. Fresh Weight of the Cultivated Wheat

**Figure 3:**
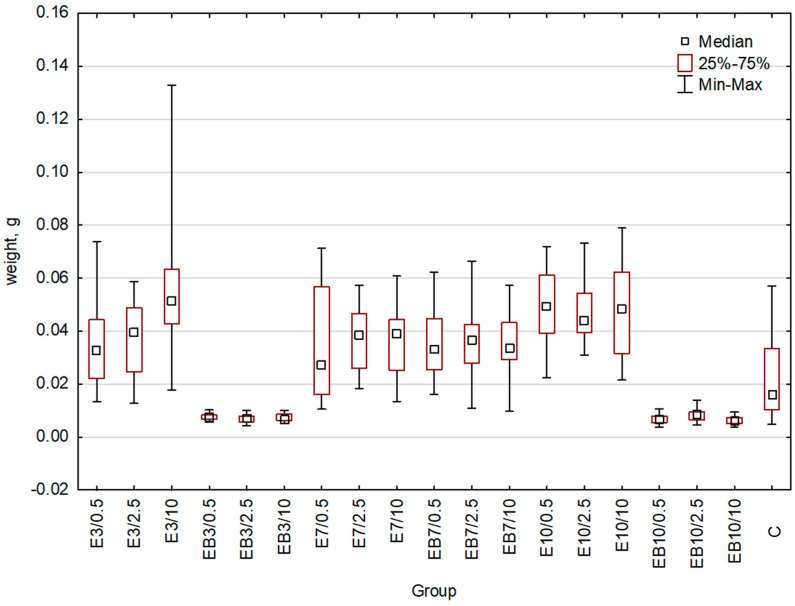
Wheat weight comparison: whiskers represent the maximum and minimum, the box represents interquartile range (25–75%), square denotes the median for the entire sample. C-control, E3-group treated with the aqueous extract obtained at 25 °C pH = 3, E7-group treated with the aqueous extract obtained at 25 °C pH = 7, E10-group treated with the aqueous extract obtained at 25 °C pH = 10, EB3-group treated with the aqueous extract obtained at 75 °C pH = 3, EB7-group treated with the aqueous extract obtained at 75 °C pH = 7, EB10-group treated with the aqueous extract obtained at 75 °C pH = 10. Concentrations used: 0.5-concentration 0.5%, 2.5-concentration 2.5%, 10-concentration 10%.

The weight of plants can be used as a second parameter of growth. For this reason, in addition to the previously performed measurement of plant length, we weighted wheat seedlings from each group treated with differently obtained extracts. As the control, we used the group of plants treated with water (Figure 3, Table 2). An indication of statistically significant differences between the studied groups is shown in Table 2. Additional data important for statistical analysis are presented in the Supplementary Materials (Tables S4–S6). The fresh weight of plants treated with E3, E7, E10, and EB7 extracts was higher or at least similar to that of the control group (Figure 3). In the case of the EB3 and EB10 extracts, the weight was significantly decreased (respectively 65% and 72%) in comparison to the control group. Importantly, a 2-fold increase in the fresh weight of wheat seedlings treated with the 10% concentration of E3 was observed.

#### 2.1.3. Chlorophyll Content in the Cultivated Wheat

To evaluate the quality of wheat seedlings treated with *B. bassiana* extracts, chlorophyll content was determined (Table 3). For all dilutions of EB3, EB7, and EB10 extracts, higher values of total chlorophyll *Chl a* and *Chl b* were observed. The chlorophyll content increased with the concentration of the respective extract (0.5 < 2.5 < 10%). In most examined cases, chlorophyll content was higher than in the control group, except for plants treated with E3 at a concentration of 10%. The role of *Chl a* in photosynthesis is preeminent over *Chl b*, which has a supporting pigment role. In all test plants, the value was above 2, indicating a good ratio of both chlorophylls [27].

### 2.2. Multi-Elemental Composition of B. bassiana Water Extracts

The mineral composition of the tested extracts is shown in Table 4. Especially interesting is the very high content of potassium (about 400 mg/L), phosphorus (over 30 mg/L), and sulfur (over 20 mg/L) in all tested extracts. In extracts, we also detected other macroelements, such as calcium, magnesium, and sodium. Among the wide spectrum of microelements detected in extracts were boron, cobalt, chromium, cuprum, ferrum, molybdenium, nickel, and zinc. Trace amounts of toxic metals, especially cadmium, were present.

### 2.3. Evaluation of Antibacterial Activity of B. bassiana Extracts

To test possible interferences between microorganisms, we evaluated the antibacterial activity of *B. bassiana* extracts against a Gram-positive bacteria strain (*Bacillus subtilis*) and Gram-negative bacteria strain (*Escherichia coli*) using the agar disc diffusion method. After 24 h of incubation, we observed no inhibition zone for the tested extracts, demonstrating the lack of antibacterial activity of the tested extracts. 

## 3. Discussion

Recent studies showed that, in addition to their classical function as insects’ pathogens, entomopathogenic fungi exert beneficial effects as stimulators of plant growth (leading to shoot growth, more leaves, higher fresh weight) and protectants against infection. Unfortunately, the efficiency of entomopathogenic fungi is dependent on the different environmental factors, especially temperature, humidity, UV solar radiation, and rain [28].

Previous tests were performed with *B. bassiana* conidia suspension added to soil or hydroponic culture, or alternatively, seed coating methods with fungal spores were used [29,30]. Considering the documented beneficial interactions of *B. bassiana* with a wide range of plants, we aimed to test possibility of using aqueous extracts of the fungus, as a safe and easy-to-obtain material, to stimulate the growth of an agriculturally significant plant—wheat. Extracts obtained from fungal biomass are known sources of biomolecules presenting diverse activities [31,32].

The use of aqueous extracts of *B. bassiana* biomass may be a positive alternative that will not cause public concern about the presence of spores in the plant and possible effects on human health [33,34]. Additionally, extracts are more stable in comparison to fungi and can be used in different environmental conditions. However, to date, entomopathogenic fungi extracts have not been tested as potential stimulants of plant growth, though macroalgae (*Ulva linza* and *Corallina officinalis)* extracts were successfully utilized for stimulating the growth and production of a range of plants. The studies showed that the concentration of extracts highly affected the results of experiments [35]. The composition of extract is affected by the preparation conditions, including temperature, solvents used for extraction, and time of contact with solvent [36]. Nikkilä et al. used heat-inactivated fungal biomass from industrial enzyme production to extract biopolymers using aqueous and alkaline methods. Aqueous extraction resulted in a higher yield of amino acids (61.3%), while alkaline extraction yielded only 27.6% amino acids [37].

In our study, the highest increase in plant length was obtained after applying extracts (E3 and E10) obtained at room temperature and used at 10% concentration (43% and 51% increase in comparison to the control group). The increase was comparable to that obtained using arbuscular mycorrhizal fungi [38], *Pseudomonas fluorescens* [39], or marine yeast [40]. The best stimulation activity of the 10% E3 extract was supported by a 2-fold increase in the fresh weight of seedlings treated with this extract. Additionally, in this case, results were comparable to those previously documented for arbuscular mycorrhizal fungi [41], *Penicillium bilaji* [42], and *Rhizobium* spp. [43]. We assume that the low extraction temperature allowed the bioactive molecules originally present in the fungal biomass to be preserved, while the use of high temperatures could have led to the degradation of some of these compounds.

For proper growth and reproduction, plants need adequate amounts of nutrients, which are necessary for a balanced plant metabolism and needed throughout their life cycle. The required micro- and macroelements, including nitrogen, calcium potassium, phosphorus, sulfur, magnesium, iron, boron, manganese, zinc, copper, nickel, and molybdenum, can be delivered from the soil [44]. Unfortunately, intensive cultivation of crops results in the removal of such nutrients every year, and the reserves of each element are depleted [35]. The effects of plant nutrient deficiencies can be manifested in various ways: inhibition of the plant’s growth dynamics, deformation of the plant, the appearance of pigmentation, or complete inhibition of plant growth [45]. Nitrogen is extremely important for plant growth, and the form of ammonium nitrogen present in tested extracts is extremely important as an easily assimilable form of nitrogen for plants [46]. Our data show that *B. bassiana* extracts contain macro- and micronutrients necessary for proper plant growth.

Chlorophyll is an important photosynthetic pigment involved in the photosynthetic reaction, which is the most important source of energy for plant growth [47]. *Chl a* and *Chl b* are involved in the absorption of solar light and are necessary for the initial reaction of photosynthesis. Therefore, the sum of *Chl a* and *Chl b* and the ratio of *Chl a*/*Chl b* are directly related to the plant’s ability to carry out photosynthesis [27,48]. We hypothesize that the high concentrations of chlorophyll in wheat seedlings treated with extracts obtained at high temperatures independent of the pH conditions (EB3, EB7, and EB10) is related to the high content of Mg and Fe detected in these extracts. Both minerals were shown to be crucial for photosynthesis, and can affect chlorophyll synthesis [49,50].

Microorganisms are important components of the soil ecosystem. They participate in providing plants with nutrients [51], stimulate plant growth, and control the activity of plant pathogens [52]. Microorganisms also contribute to the improvement in soil structure, fertility, and porosity, all of which are linked to agronomic productivity. The ability of soil bacteria to bioremediate contaminated soils, i.e., mineralization of organic pollutants, is also known [53,54]. The introduction of antibacterial substances into the soil together with fertilizers could lead to an imbalance between the plant and soil bacteria, which could result in the deterioration of plant growth. We showed that *B. bassiana* water extracts do not pose a threat to these beneficial microorganisms. In addition, the mineral composition of tested *extracts* revealed the presence of several nutrients. Enhancement of the tested wheat’s growth seems to be related to the composition of the extracts, which we documented as a rich source of macro- and microelements.

## 4. Materials and Methods

### 4.1. Chemicals

All the reagents used in the experiment were of an analytical-grade purity. Ammonia solution, nitric acid, methanol, ethanol, and acetone were purchased from POCH SA (Gliwice, Poland). Water was deionized with Integrated Merck/Millipore ultrapure and pure water System (Merck KGaA, Darmstadt, Germany).

### 4.2. Fungi Cultivation

The *B. bassiana* strain was a kind gift from prof. Cezary Tkaczuk from Siedlce University of Natural Sciences and Humanities (Siedlce, Poland). Fungus was cultivated in Potato Dextrose Broth (PDB) medium (A&A Biotechnology, Gdansk, Poland). First, a pre-culture was made by transferring the spores with a piece of solid PDB medium (1 cm^2^ slice), containing 2% of agar (Merck KGaA, Darmstadt, Germany), into an Erlenmeyer flask with 100 mL of PDB medium. Then, culture was incubated for 7 days on a rotary shaker (PSU-21, Biosan, Riga, Latvia) at 150 rpm at 25 °C. After 7 days, 4 mL of inoculum was transferred to other flasks and cultures were incubated in the same conditions (150 rpm, 25 °C) for the next 13 days. Later, the biomass was separated from the liquid medium by vacuum filtration using a Büchner funnel. The biomass was washed 3 times with 100 mL of deionized water and then dried at 50 °C for 78 h.

### 4.3. Extract Preparation

Selected dilutions (0.5%, 2.5%, or 10%) of the crude extract obtained under different pH (3, 7, or 10) and temperature (25 °C or 75 °C) conditions were used to establish optimal method of extraction. Extraction of mycelium was performed in two ways based on the modified procedure described by Godlewska et al. [55]. In the first method, the dried and milled fungal biomass (0.2 g) was added to 10 mL of deionized water with pH 3, 7, and 10 (extracts marked as E3, E7, and E10, respectively). To set the appropriate pH (using pH meter Seven Multi; Mettler Toledo, Greifensee, Switzerland), ammonia solution and nitric acid were used. Next, the flasks were incubated on a magnetic stirrer for 1 h at 25 °C. The second method was a variation of the previous one with the addition of a heating step; 0.2 g of dried and milled fungal biomass was added to 10 mL of deionized water with the pH set to 3, 7, and 10 (extracts marked as EB3, EB7, and EB10, respectively). Then, samples were incubated for 1 h on a magnetic stirrer at 75 °C. After extraction, all samples were filtered using Nylon 0.2 mm filters (Equimed, Cracow, Poland). The obtained supernatants were set as a 100% fungal liquid extract.

### 4.4. Multi-Elemental Composition of Fungal Extracts

Sample mineralization was carried out with a microwave digestion system (Start D, Milestone) followed by multi-elemental analysis with an inductively coupled plasma optical emission spectrometer (ICP-OES iCAP Duo Thermo Scientific, Thermo Fisher Scientific, Waltham, MA, USA). Content of N-NH_4_^+^ was determined according to the EPA 350.2 method. 

### 4.5. Antibacterial Assays

To evaluate the antimicrobial activity of extracts, the agar disc diffusion test (diffusion method) was performed. *E. coli* (ATCC 25922, American Type Culture Collection, Gaithersburg, MD, USA) representing Gram-negative bacteria, and *B. subtilis* (ATCC 6633, ATCC, Gaithersburg, MD, USA), representing Gram-positive bacteria, were grown on Mueller–Hinton agar (Sigma Aldrich, St. Luis, MO, USA) plates for 24 h. Then, biomass from one or two bacterial colonies was collected and suspended in a tube containing sterile saline. The turbidity of the bacterial suspensions (expressed as optical density, OD) was measured using an optical spectrophotometer (Varian Cary 50 Conc. Instrument, Varian Inc., Victoria, Australia) (λ = 600 nm) and adjusted to 0.25 with sterile saline. A total of 50 μL of the bacterial cell suspension was dispensed and spread onto agar plates. Then, filter paper discs (6 mm in diameter) were individually impregnated with 10 µL of the tested extracts. After 2 h of drying on air, the discs were placed on the plates with bacterial cultures. After 24 h of incubation at 37 °C, the influence of the fungal extracts on the growth of both tested bacteria was assessed. A toxic effect after exposure of the bacteria to the tested extracts was determined by measuring the diameter of the zone of inhibition in millimeters. The experiments were carried out in triplicate.

### 4.6. Germination Tests

The growth-stimulating activity of the different concentrations of fungal extracts was evaluated by the wheat germination test. For each group, tests in standardized conditions using a Jacobsen apparatus according to the International Seed Testing Association (ISTA) were performed. Twenty seeds of wheat (Wheat Artist, HR Smolice, Smolice, Poland) were aseptically placed at equal intervals on sterile Petri dishes lined with cotton wool. After seed stratification (24 h, 4 °C), each Petri dish was moistened with 10 mL of distilled water. After 3 days, all dishes were treated with 5 mL of different concentrations of tested extracts (samples) or water (control). The seed germination was run for 14 days at 22 °C in the day–night mode (16 h:8 h). After 14 days, the effect of fungal extracts on wheat growth was determined by measuring the plant’s length and weight. The experiments were performed in triplicate.

### 4.7. Chlorophyll Content in Cultivated Plants

To test the impact of fungal extracts on the chlorophyll content, the fresh green parts of the wheat cultivated without extracts addition (control) and with addition of different extracts (samples) were used. The total chlorophyll (*Chl tot*), chlorophyll a (*Chl a*), and chlorophyll b (*Chl b*) level/amount in the extracts from the fresh green parts of wheat was determined by measuring the absorbance at the 663 nm and 645 nm wavelengths using UV-Vis spectrophotometer (Varian Cary 50 Conc. Instrument, Victoria, Australia). The extracts of chlorophylls were prepared in 80% acetone. The chlorophyll content was calculated using Equations (1)–(3) [56]:

Chl tot = 8.02∙Abs(663) + 20.2∙Abs(645);(1)

Chl a = 12.7∙Abs(663) − 2.69∙Abs(645);(2)

Chl b = 22.9∙Abs(645) − 4.68∙Abs(663)(3)

where Abs(663), Abs(645), and Abs(663) are the absorbances for wavelengths 663 nm, 645 nm, and 663 nm, respectively.

### 4.8. Statistical Analysis

The results were elaborated statistically by Statistica ver. 13 (StatSoft Polska Sp. Zoo, Cracow, Poland). Normality of the experimental results distribution was assessed using the Shapiro–Wilk test. For a distribution other than normal, the Kruskal–Wallis test was used. Results were considered significantly different when *p* < 0.05.

## 5. Conclusions

Entomopathogenic fungi, such as *B. bassiana*, represent a unique source of alternative chemical fertilizers. In the present study, the stimulating effect of aqueous extracts from *B. bassiana* on the growth of tested plants was demonstrated. We documented that the obtained extracts contained micro- and macroelements indispensable for plant growth and expansion. Tested extracts presented no antibacterial activity, which suggests that they would not exert an adverse effect on the soil bacteria and environment. Importantly, our study presents for the first time the positive impact of fungal extracts on plant growth. Advantageously, our results show that the highest growth-promoting activity was presented by extracts obtained at room temperature. It is worth mentioning that using water for the extraction process is an additional benefit. Our study is preliminary and additional research needs to be carried out to optimize the extract dissolution ratio to confirm the effects in real environment conditions and to check the consistency of the plant growth stimulation across different plant species. However, we believe that our results will be a stimulus and a starting point for further research in this direction.

## Figures and Tables

**Table 1:** The table shows the length of the green part of cultivated wheat treated with the fungal extracts. Index *—indicates the presence of statistically significant differences between the control and tested groups.

Sample Name	Extraction Temperature	Extraction pH	Concentration of the Extract	Length of Cultivated Wheat
	°C	-	%	cm
Control	-	-	-	7.92
E3/0.5	25	3	0.5	9.57
E3/2.5	25	3	2.5	9.99 *
E3/10	25	3	10	11.33 *
EB3/0.5	75	3	0.5	7.46
EB3/2.5	75	3	2.5	7.94
EB3/10	75	3	10	7.39
E7/0.5	25	7	0.5	11.27 *
E7/2.5	25	7	2.5	10.80 *
E7/10	25	7	10	10.24 *
EB7/0.5	75	7	0.5	9.95 *
EB7/2.5	75	7	2.5	10.42 *
EB7/10	75	7	10	10.42 *
E10/0.5	25	10	0.5	11.66 *
E10/2.5	25	10	2.5	11.47 *
E10/10	25	10	10	11.99 *
EB10/0.5	75	10	0.5	7.52
EB10/2.5	75	10	2.5	8.90
EB10/10	75	10	10	5.45

**Table 2:** Fresh mass of the wheat treated with *B. bassiana* mycelium water extracts. Index *—indicates the presence of statistically significant differences between the control and tested groups.

Sample Name	Extraction Temperature	Extraction pH	Concentration of the Extract	Fresh Mass
	°C	-	%	g
Control	-	-	-	0.02230
E3/0.5	25	3	0.5	0.03477
E3/2.5	25	3	2.5	0.03708
E3/10	25	3	10	0.05058 *
EB3/0.5	75	3	0.5	0.00777
EB3/2.5	75	3	2.5	0.00702 *
EB3/10	75	3	10	0.00723 *
E7/0.5	25	7	0.5	0.03519
E7/2.5	25	7	2.5	0.03719
E7/10	25	7	10	0.03487
EB7/0.5	75	7	0.5	0.03530
EB7/2.5	75	7	2.5	0.03593
EB7/10	75	7	10	0.03418
E10/0.5	25	10	0.5	0.04996 *
E10/2.5	25	10	2.5	0.04598 *
E10/10	25	10	10	0.04700 *
EB10/0.5	75	10	0.5	0.00672 *
EB10/2.5	75	10	2.5	0.00827
EB10/10	75	10	10	0.00622 *

**Table 3:** Evaluation of chlorophyll content in the tested wheat treated with fungal extracts. C—control, E3—group treated with the aqueous extract obtained at 25 °C pH = 3, E7—group treated with the aqueous extract obtained at 25 °C pH = 7, E10—group treated with the aqueous extract obtained at 25 °C pH = 10, EB3—group treated with the aqueous extract obtained at 75 °C pH = 3, EB7—group treated with the aqueous extract obtained at 75 °C pH = 7, EB10—group treated with the aqueous extract obtained at 75 °C pH = 10. Concentrations used: 0.5—concentration 0.5%, 2.5—concentration 2.5%, 10—concentration 10%.

Sample	*Chl a*	*Chl b*	*Chl tot*	*Chl a/Chl b*
	mg/L	mg/L	mg/L	-
K	631.2 ± 7.19	256.5 ± 1.41	887.5 ± 3.42	2.46
E3/0.5	971.3 ± 8.11	361.6 ± 2.11	1333 ± 7.10	2.69
E3/2.5	750.3 ± 9.12	270.1 ± 1.09	1020 ± 8.29	2.78
E3/10	562.5 ± 7.41	227.2 ± 2.17	789.5 ± 7.41	2.48
E7/0.5	1026 ± 4.19	320.5 ± 1.01	1346 ± 6.06	3.20
E7/2.5	1084 ± 5.22	414.7 ± 4.13	1498 ± 6.12	2.61
E7/10	1329 ± 4.77	510.6 ± 4.09	1839 ± 7.14	2.60
E10/0.5	959.2 ± 4.13	409.3 ± 2.19	1368 ± 5.16	2.34
E10/2.5	821.1 ± 7.12	316.3 ± 1.02	1137 ± 9.11	2.60
E10/10	582.2 ± 6.36	262.2 ± 0.98	844.2 ± 5.09	2.22
EB3/0.5	2334 ± 10.14	825.3 ± 2.33	3159 ± 12.32	2.83
EB3/2.5	2624 ± 9.79	860.5 ± 2.54	3483 ± 11.19	3.05
EB3/10	2851 ± 9.65	934.7 ± 1.45	3785 ± 10.09	3.05
EB7/0.5	1038 ± 10.46	399.5 ± 2.19	1437 ± 9.06	2.60
EB7/2.5	810.7 ± 4.15	288.3 ± 1.12	1099 ± 4.96	2.81
EB7/10	805.3 ± 3.96	279.1 ± 0.76	1084 ± 7.44	2.89
EB10/0.5	1767 ± 5.57	560.0 ± 3.04	2327 ± 5.11	3.16
EB10/2.5	2517 ± 4.97	863.7 ± 2.71	3379 ± 6.29	2.91
EB10/10	1575 ± 11.63	553.9 ± 1.22	2128 ± 11.09	2.84

**Table 4:** The elemental composition of *B. bassiana* extracts. The table presents the content of micronutrients, macronutrients, toxic metals, and ammonia nitrogen in the tested extracts (E3—aqueous extract obtained at 25 °C pH = 3, EB3—aqueous extract obtained at 75 °C pH = 3, E7—aqueous extract obtained at 25 °C pH = 7, EB7—aqueous extract obtained at 75 °C pH = 7, E10—aqueous extract obtained at 25 °C pH = 10, EB10—aqueous extract obtained at 75 °C pH = 10).

Element	E3	EB3	E7	EB7	E10	EB10
	mg/L	mg/L	mg/L	mg/L	mg/L	mg/L
Macroelements						
Ca	5.317 ± 0.204	5.140 ± 0.132	6.613 ± 0.221	6.923 ± 0.169	3.863 ± 0.092	7.047 ± 0.172
K	411.3 ± 21.1	389.1 ± 19.23	463.1 ± 17.22	484.6 ± 23.56	470.1 ± 28.63	409.9 ± 22.39
Mg	11.11 ± 0.43	14.18 ± 0.59	8.469 ± 0.298	15.21 ± 0.66	12.41 ± 0.36	15.02 ± 0.24
Na	9.875 ± 0.33	7.057 ± 0.19	12.40 ± 48	10.22 ± 0.31	9.927 ± 0.22	9.52 ± 0.19
P	34.33 ± 1.09	45.56 ± 1.22	27.26 ± 0.41	52.04 ± 1.17	50.76 ± 1.66	44.23 ± 0.97
S	23.54 ± 0.96	20.35 ± 1.27	27.00 ± 1.11	30.88 ± 0.99	29.35 ± 1.19	22.43 ± 1.31
Microelements						
B	0.275 ± 0.013	0.119 ± 0.022	0.159 ± 0.009	0.082 ± 0.007	0.050 ± 0.006	0.048 ± 0.009
Co	0.0015 ± 0.0002	0.0065 ± 0.0004	0.0092 ± 0.0003	<LOD	<LOD	<LOD
Cr	0.031 ± 0.004	0.271 ± 0.014	0.026 ± 0.005	0.524 ± 0.011	0.025 ± 0.002	0.022 ± 0.004
Cu	0.190 ± 0.013	0.179 ± 0.009	0.197 ± 0.011	0.202 ± 0.019	0.275 ± 0.023	0.236 ± 0.017
Fe	0.789 ± 0.94	1.329 ± 0.097	0.605 ± 0.033	2.651 ± 0.184	0.753 ± 0.037	0.759 ± 0.041
Mn	0.018 ± 0.002	0.043 ± 0.004	0.030 ± 0.002	0.053 ± 0.003	0.020 ± 0.001	0.031 ± 0.001
Mo	0.025 ± 0.001	<LOD	0.038 ± 0.002	0.062 ± 0.001	0.081 ± 0.003	0.032 ± 0.001
Ni	0.112 ± 0.004	0.228 ± 0.009	0.091 ± 0.001	0.320 ± 0.011	0.051 ± 0.001	0.072 ± 0.004
Zn	0.197 ± 0.007	0.165 ± 0.011	0.308 ± 0.024	0.096 ± 0.005	0.116 ± 0.007	0.115 ± 0.009
Toxic metals						
As	<LOD	<LOD	0.109 ± 0.0019	0.310 ± 0.042	<LOD	<LOD
Cd	0.0043 ± 0.0002	0.0101 ± 0.0004	0.0080 ± 0.0002	0.0101 ± 0.0004	0.0071 ± 0.0005	0.0117 ± 0.0009
Pb	0.093 ± 0.005	0.045 ± 0.004	0.057 ± 0.004	<LOD	<LOD	0.064 ± 0.002
Ammonia nitrogen						
N-NH_3_	133.07 ± 11.35	27.22 ± 4.22	139.60 ± 9.46	85.53 ± 7.13	146.17 ± 8.69	64.13 ± 3.11

<LOD below the Limit of Detection.

## Data Availability

Data is contained within the article and Supplementary Materials to the article.

## Supplementary Materials

The following supporting information can be downloaded at: https://www.mdpi.com/article/10.3390/plants12020326/s1, Table S1: The results of tested plants length measurements; Table S2: The Shapiro-Wilk test p-values for plants length measurement. Table S3: The Kruskal-Wallis test p-values for plants length measurement. Table S4: The results of tested plants fresh weight measurements; Table S5: The Shapiro-Wilk test p-values for plants fresh weight measurement. Table S6: The Kruskal-Wallis test p-values for plants fresh weight measurement.
