# Supplementary material for: Beauveria bassiana Water Extracts’ Effect on the Growth of Wheat
Source: Plants (Basel). 2023 Jan 10;12(2):326. doi: 10.3390/plants12020326 (PMC9863656; doi:10.3390/plants12020326)
Supplement: Supplementary file 1 [file plants-12-00326-s001.zip › plants-2073226-supplementary.pdf]

Beauveria bassiana Water Extracts' effect on the Growth of Wheat

Dawid J. Kramski [1,2,5], Daria Nowinski [3,5], Kaja Kowalczuk [5], Piotr Kruszyński [5], Jagoda Radzimska [5] and Beata Greb-Markiewicz [4,*]

1 Department of Advanced Material Technology, Faculty of Chemistry, Wroclaw University of Science and Technology, Wybrzeze Wyspianskiego 27, 50-370 Wroclaw, Poland; dawid.kramski@pwr.edu.pl
2 Department of Analytical Chemistry and Chemical Metallurgy, Faculty of Chemistry, Wroclaw University of Science and Technology, Wybrzeze Wyspianskiego 27, 50-370 Wroclaw, Poland
3 Department of Organic and Organic and Medicinal Chemistry, Faculty of Chemistry, Wroclaw University of Science and Technology, Wybrzeze Wyspianskiego 27, 50-370 Wroclaw, Poland; daria.kocek@pwr.edu.pl
4 Department of Biochemistry, Molecular Biology and Biotechnology, Faculty of Chemistry, Wroclaw University of Science and Technology, Wybrzeze Wyspianskiego 27, 50-370 Wroclaw, Poland
5 Students Science Association Bio-Top, Faculty of Chemistry, Wroclaw University of Science and Technology, Wybrzeze Wyspianskiego 27, 50-370 Wroclaw, Poland; 252353@student.pwr.edu.pl (K.K.); 252359@student.pwr.edu.pl (P.K.); 258605@student.pwr.edu.pl (J.R.)
* Correspondence: beata.greb-markiewicz@pwr.edu.pl

Supplementary Information for Statistical Analysis:

Table S1. Results of the length measuring with means, standard deviation, standard deviation error, median and quartiles (Q25 and Q75). Shapiro-Wilk test if p-value > 0.05 there is a normal distribution. p-values for the Shapiro-Wilk test are shown in the **Table S2**. Since the distribution is other than normal for one group, the Kruskal-Wallis test was used in the next step. The p-values for the Kruskal-Wallis test are shown in the **Table S3**.

Table S4. Results for the fresh weight measurement with means, standard deviation, standard deviation error, median and quartiles (Q25 and Q75). Shapiro-Wilk test if p-value > 0.05 there is a normal distribution. p-values for the Shapiro-Wilk test are shown in the **Table S5**. Since the distribution is other than normal for one group, the Kruskal-Wallis test was used in the next step. The p-values for the Kruskal-Wallis test are shown in the **Table S6**.

Table S1. The results of tested plants length measurement. In the Table means, standard deviation, standard deviation error, median and quartiles (Q25 and Q75) are presented.

Group	Means	Std.Dev.	Std.Err.	Q25	Median	Q75
E3/0.5	9,57	1,62	0,29	8,05	9,75	10,85
E3/2.5	9,99	1,78	0,30	8,20	10,10	11,40
E3/10	11,33	2,32	0,40	9,60	11,50	13,00
EB3/0.5	7,46	0,80	0,16	6,80	7,40	8,00
EB3/2.5	7,94	0,98	0,20	7,20	8,00	8,60
EB3/10	7,39	1,10	0,22	6,40	7,30	8,00
E7/0,5	11,27	1,80	0,30	10,00	11,10	12,60
E7/2,5	10,80	1,44	0,23	10,00	10,70	12,00
E7/10	10,24	2,00	0,32	7,90	11,05	11,70
EB7/0.5	9,95	1,62	0,27	8,60	10,00	11,30

EB7/2.5	10,42	1,58	0,27	8,80	10,80	11,60
EB7/10	10,42	1,25	0,21	9,50	10,60	11,40
E10/0.5	11,66	1,80	0,30	10,20	11,70	13,00
E10/2.5	11,47	1,21	0,20	10,50	11,55	12,40
E10/10	11,99	2,03	0,33	9,70	12,20	13,70
EB10/0.5	7,52	1,39	0,27	6,10	7,30	8,80
EB10/2.5	8,90	1,11	0,22	8,00	9,05	9,90
EB10/10	5,45	0,93	0,19	4,90	5,55	5,95
C	7,92	1,59	0,24	6,90	8,10	9,10

Table S2. The Shapiro-Wilk test p-values for tested plants length measurement.

group	p-value
E3/0.5	0.0831
E3/2.5	0.0861
E3/10	0.5198
E7/0.5	0,5713
E7/2.5	0,5715
E7/10	0,0006
E10/0.5	0,0358
E10/2.5	0,7064
E10/10	0,0267
EB3/0.5	0,5396
EB3/2.5	0,8751
EB3/10	0,1020
EB7/0.5	0,0878
EB7/2.5	0,0314
EB7/10	0,3903
EB10/0.5	0,0997
EB10/2.5	0,1193
EB10/10	0,9751
Control	0,3429

Table S3. The Kruskal-Wallis test p-values for tested plants length measurement.

	E3/0.5	E3/2.5	E3/10	EB3/0.5	EB3/2.5	EB3/10	E7/0,5	E7/2,5	E7/10	EB7/0.5	EB7/2.5	EB7/10	E10/0.5	E10/2.5	E10/10	EB10/0.5	EB10/2.5	EB10/10	C
E3/0.5		1,000000	0,590318	0,033077	0,610244	0,033739	0,417228	1,000000	1,000000	1,000000	1,000000	1,000000	0,022589	0,029654	0,006664	0,098323	1,000000	0,000005	0,405781
E3/2.5	1,000000		1,000000	0,001007	0,036694	0,001031	1,000000	1,000000	1,000000	1,000000	1,000000	1,000000	0,319432	0,420247	0,115332	0,003497	1,000000	0,000000	0,012962
E3/10	0,590318	1,000000		0,000000	0,000002	0,000000	1,000000	1,000000	1,000000	1,000000	1,000000	1,000000	1,000000	1,000000	1,000000	0,000000	0,009648	0,000000	0,000000
EB3/0.5	0,033077	0,001007	0,000000		1,000000	1,000000	0,000000	0,000000	0,000049	0,001244	0,000017	0,000015	0,000000	0,000000	0,000000	1,000000	1,000000	1,000000	1,000000
EB3/2.5	0,610244	0,036694	0,000002	1,000000		1,000000	0,000001	0,000020	0,003056	0,043761	0,001169	0,001040	0,000000	0,000000	0,000000	1,000000	1,000000	1,000000	1,000000
EB3/10	0,033739	0,001031	0,000000	1,000000	1,000000		0,000000	0,000000	0,000050	0,001274	0,000018	0,000015	0,000000	0,000000	0,000000	1,000000	1,000000	1,000000	1,000000
E7/0,5	0,417228	1,000000	1,000000	0,000000	0,000001	0,000000		1,000000	1,000000	1,000000	1,000000	1,000000	1,000000	1,000000	1,000000	0,000000	0,005669	0,000000	0,000000
E7/2,5	1,000000	1,000000	1,000000	0,000000	0,000020	0,000000	1,000000		1,000000	1,000000	1,000000	1,000000	1,000000	1,000000	1,000000	0,000001	0,053403	0,000000	0,000001
E7/10	1,000000	1,000000	1,000000	0,000049	0,003056	0,000050	1,000000	1,000000		1,000000	1,000000	1,000000	1,000000	1,000000	0,671352	0,000187	1,000000	0,000000	0,000554
EB7/0.5	1,000000	1,000000	1,000000	0,001244	0,043761	0,001274	1,000000	1,000000	1,000000		1,000000	1,000000	0,270091	0,355476	0,095983	0,004291	1,000000	0,000000	0,016071
EB7/2.5	1,000000	1,000000	1,000000	0,000017	0,001169	0,000018	1,000000	1,000000	1,000000	1,000000		1,000000	1,000000	1,000000	1,000000	0,000068	0,789296	0,000000	0,000194
EB7/10	1,000000	1,000000	1,000000	0,000015	0,001040	0,000015	1,000000	1,000000	1,000000	1,000000	1,000000		1,000000	1,000000	1,000000	0,000058	0,752911	0,000000	0,000161
E10/0.5	0,022589	0,319432	1,000000	0,000000	0,000000	0,000000	1,000000	1,000000	1,000000	0,270091	1,000000	1,000000		1,000000	1,000000	0,000000	0,000174	0,000000	0,000000
E10/2.5	0,029654	0,420247	1,000000	0,000000	0,000000	0,000000	1,000000	1,000000	1,000000	0,355476	1,000000	1,000000	1,000000		1,000000	0,000000	0,000224	0,000000	0,000000
E10/10	0,006664	0,115332	1,000000	0,000000	0,000000	0,000000	1,000000	1,000000	0,671352	0,095983	1,000000	1,000000	1,000000	1,000000		0,000000	0,000040	0,000000	0,000000
EB10/0.5	0,098323	0,003497	0,000000	1,000000	1,000000	1,000000	0,000000	0,000001	0,000187	0,004291	0,000068	0,000058	0,000000	0,000000	0,000000		1,000000	1,000000	1,000000
EB10/2.5	1,000000	1,000000	0,009648	1,000000	1,000000	1,000000	0,005669	0,053403	1,000000	1,000000	0,789296	0,752911	0,000174	0,000224	0,000040	1,000000		0,006267	1,000000
EB10/10	0,000005	0,000000	0,000000	1,000000	1,000000	1,000000	0,000000	0,000000	0,000000	0,000000	0,000000	0,000000	0,000000	0,000000	0,000000	1,000000	0,006267		0,332807
Control	0,405781	0,012962	0,000000	1,000000	1,000000	1,000000	0,000000	0,000001	0,000554	0,016071	0,000194	0,000161	0,000000	0,000000	0,000000	1,000000	1,000000	0,332807	

Table S4. The results of tested plants fresh weight measurement. In the Table means, standard deviation, standard deviation error, median and quartiles (Q25 and Q75) are presented.

Group	Means	Std.Dev.	Std.Err.	Q25	Median	Q75
E3/0.5	0,034772	0,015529	0,002745	0,022150	0,032850	0,044300
E3/2.5	0,037077	0,013533	0,002287	0,024700	0,039500	0,048700
E3/10	0,054212	0,020834	0,003627	0,042700	0,051200	0,063400
EB3/0.5	0,007772	0,001155	0,000231	0,006800	0,007800	0,008300
EB3/2.5	0,007020	0,001483	0,000297	0,005800	0,007300	0,008000
EB3/10	0,007228	0,001463	0,000293	0,006300	0,006700	0,008600
E7/0,5	0,035195	0,021481	0,003531	0,016100	0,027400	0,056800
E7/2,5	0,037187	0,012144	0,001970	0,026000	0,038750	0,046500
E7/10	0,034871	0,012556	0,002037	0,025300	0,039200	0,044400
EB7/0.5	0,035303	0,011919	0,002015	0,025500	0,033100	0,044800
EB7/2.5	0,035929	0,013582	0,002329	0,027800	0,036700	0,042500
EB7/10	0,034183	0,012472	0,002108	0,029200	0,033800	0,043200
E10/0.5	0,049957	0,013441	0,002272	0,039200	0,049300	0,061200
E10/2.5	0,045984	0,009922	0,001610	0,039600	0,044100	0,054200
E10/10	0,047000	0,017850	0,002935	0,031600	0,048600	0,062300
EB10/0.5	0,006719	0,001670	0,000321	0,005400	0,006600	0,007800
EB10/2.5	0,008273	0,002122	0,000416	0,006500	0,008600	0,009600
EB10/10	0,006217	0,001508	0,000308	0,005050	0,006250	0,007350
Control	0,022295	0,015690	0,002393	0,010400	0,015800	0,033500

Table S5. The Shapiro-Wilk test p-values for plants fresh weight measurement.

group	p-value
E3/0.5	0.0877
E3/2.5	0.0684
E3/10	0.0026
E7/0.5	0,0002
E7/2.5	0,0385
E7/10	0,0111
E10/0.5	0,3214
E10/2.5	0,0411
E10/10	0,0219
EB3/0.5	0,7548
EB3/2.5	0,9276
EB3/10	0,0295
EB7/0.5	0,3256
EB7/2.5	0,7844
EB7/10	0,367
EB10/0.5	0,8749
EB10/2.5	0,4286
EB10/10	0,5007
Control	0,2341

Table S6. The Kruskal-Wallis test p-values for plants fresh weight measurement.

	E3/0.5	E3/2.5	E3/10	EB3/0.5	EB3/2.5	EB3/10	E7/0,5	E7/2,5	E7/10	EB7/0.5	EB7/2.5	EB7/10	E10/0.5	E10/2.5	E10/10	EB10/0.5	EB10/2.5	EB10/10	C
E3/0.5		1,000000	0,315225	0,000014	0,000001	0,000002	1,000000	1,000000	1,000000	1,000000	1,000000	1,000000	0,568464	1,000000	1,000000	0,000000	0,000030	0,000000	1,000000
E3/2.5	1,000000		1,000000	0,000000	0,000000	0,000000	1,000000	1,000000	1,000000	1,000000	1,000000	1,000000	1,000000	1,000000	1,000000	0,000000	0,000001	0,000000	0,197576
E3/10	0,315225	1,000000		0,000000	0,000000	0,000000	0,218433	1,000000	0,318661	0,404678	0,552419	0,179544	1,000000	1,000000	1,000000	0,000000	0,000000	0,000000	0,000001
EB3/0.5	0,000014	0,000000	0,000000		1,000000	1,000000	0,000005	0,000000	0,000002	0,000004	0,000003	0,000013	0,000000	0,000000	0,000000	1,000000	1,000000	1,000000	0,161622
EB3/2.5	0,000001	0,000000	0,000000	1,000000		1,000000	0,000000	0,000000	0,000000	0,000000	0,000000	0,000001	0,000000	0,000000	0,000000	1,000000	1,000000	1,000000	0,023388
EB3/10	0,000002	0,000000	0,000000	1,000000	1,000000		0,000001	0,000000	0,000000	0,000000	0,000000	0,000001	0,000000	0,000000	0,000000	1,000000	1,000000	1,000000	0,034111
E7/0,5	1,000000	1,000000	0,218433	0,000005	0,000000	0,000001		1,000000	1,000000	1,000000	1,000000	1,000000	0,405369	1,000000	1,000000	0,000000	0,000012	0,000000	1,000000
E7/2,5	1,000000	1,000000	1,000000	0,000000	0,000000	0,000000	1,000000		1,000000	1,000000	1,000000	1,000000	1,000000	1,000000	1,000000	0,000000	0,000000	0,000000	0,151250
E7/10	1,000000	1,000000	0,318661	0,000002	0,000000	0,000000	1,000000	1,000000		1,000000	1,000000	1,000000	0,585306	1,000000	1,000000	0,000000	0,000005	0,000000	0,779873
EB7/0.5	1,000000	1,000000	0,404678	0,000004	0,000000	0,000000	1,000000	1,000000	1,000000		1,000000	1,000000	0,730454	1,000000	1,000000	0,000000	0,000008	0,000000	0,912713
EB7/2.5	1,000000	1,000000	0,552419	0,000003	0,000000	0,000000	1,000000	1,000000	1,000000	1,000000		1,000000	0,982465	1,000000	1,000000	0,000000	0,000007	0,000000	0,766838
EB7/10	1,000000	1,000000	0,179544	0,000013	0,000001	0,000001	1,000000	1,000000	1,000000	1,000000	1,000000		0,333372	1,000000	1,000000	0,000000	0,000027	0,000000	1,000000
E10/0.5	0,568464	1,000000	1,000000	0,000000	0,000000	0,000000	0,405369	1,000000	0,585306	0,730454	0,982465	0,333372		1,000000	1,000000	0,000000	0,000000	0,000000	0,000001
E10/2.5	1,000000	1,000000	1,000000	0,000000	0,000000	0,000000	1,000000	1,000000	1,000000	1,000000	1,000000	1,000000	1,000000		1,000000	0,000000	0,000000	0,000000	0,000045
E10/10	1,000000	1,000000	1,000000	0,000000	0,000000	0,000000	1,000000	1,000000	1,000000	1,000000	1,000000	1,000000	1,000000	1,000000		0,000000	0,000000	0,000000	0,000111
EB10/0.5	0,000000	0,000000	0,000000	1,000000	1,000000	1,000000	0,000000	0,000000	0,000000	0,000000	0,000000	0,000000	0,000000	0,000000	0,000000		1,000000	1,000000	0,007120
EB10/2.5	0,000030	0,000001	0,000000	1,000000	1,000000	1,000000	0,000012	0,000000	0,000005	0,000008	0,000007	0,000027	0,000000	0,000000	0,000000	1,000000		1,000000	0,289311
EB10/10	0,000000	0,000000	0,000000	1,000000	1,000000	1,000000	0,000000	0,000000	0,000000	0,000000	0,000000	0,000000	0,000000	0,000000	0,000000	1,000000	1,000000		0,004001
C	1,000000	0,197576	0,000001	0,161622	0,023388	0,034111	1,000000	0,151250	0,779873	0,912713	0,766838	1,000000	0,000001	0,000045	0,000111	0,007120	0,289311	0,004001	
